# Validity of the I‑FEED classification in assessing postoperative gastrointestinal impairment in patients undergoing elective lumbar spinal surgery with general anesthesia: a prospective observational study

**DOI:** 10.1186/s13741-024-00409-4

**Published:** 2024-06-03

**Authors:** Chun-Yu Wu, Chih-Jun Lai, Fu-Ren Xiao, Jen-Ting Yang, Shih-Hung Yang, Dar-Ming Lai, Fon-Yih Tsuang

**Affiliations:** 1 Department of Anesthesiology, National Taiwan University Hospital Hsinchu Branch, Hsinchu City, Taiwan; 2 Department of Anesthesiology, National Taiwan University Hospital, Taipei City, Taiwan; 3 Spine Tumor Center, National Taiwan University Hospital, Taipei City, Taiwan; 4 Division of Neurosurgery, Department of Surgery, National Taiwan University Hospital, Taipei City, 100 Taiwan; 5 Department of Health Services, University of Washington, Seattle, USA

**Keywords:** Lumbar spine, Ileus, I-FEED, Gastrointestinal impairment, Postoperative gastrointestinal impairment, Postoperative gastrointestinal dysfunction

## Abstract

**Background:**

The I-FEED classification, scored 0–8, was reported to accurately describe the clinical manifestations of gastrointestinal impairment after colorectal surgery. Therefore, it is interesting to determine whether the I-FEED scoring system is also applicable to patients undergoing lumbar spine surgery.

**Methods:**

Adult patients undergoing elective lumbar spine surgery were enrolled, and the I-FEED score was measured for 4 days after surgery. The I-FEED scoring system incorporates five elements: intake (score: 0, 1, 3), feeling nauseated (score: 0, 1, 3), emesis (score: 0, 1, 3), results of physical exam (score: 0, 1, 3), and duration of symptoms (score: 0, 1, 2). Daily I-FEED scores were summed, and the highest overall score is used to categorize patients into one of three categories: normal (0–2 points), postoperative gastrointestinal intolerance (POGI; 3–5 points), and postoperative gastrointestinal dysfunction (POGD; 6 + points). The construct validity hypothesis testing determines whether the I-FEED category is consistent with objective clinical findings relevant to gastrointestinal impairment, namely, the longer length of hospital stay (LOS), higher inhospital medical cost, more postoperative gastrointestinal medical treatment, and more postoperative non-gastrointestinal complications.

**Results:**

A total of 156 patients were enrolled, and 25.0% of patients were categorized as normal, 49.4% POGI, and 25.6% POGD. Patients with higher I-FEED scores agreed with the four validity hypotheses. Patients with POGD had a significantly longer length of hospital stay (1 day longer median stay; *p* = 0.049) and more inhospital medical costs (approximately 500 Taiwanese dollars; *p* = 0.037), and more patients with POGD required rectal laxatives (10.3% vs. 32.5% vs. 32.5%; *p* = 0.026). In addition, more patients with POGD had non-gastrointestinal complications (5.1% vs. 11.7% vs. 30.0%; *p* = 0.034).

**Conclusion:**

This study contributes preliminary validity evidence for the I-FEED score as a measure for postoperative gastrointestinal impairment after elective lumbar spine surgery.

## Introduction

Postoperative ileus is a recognized complication after lumbar spine surgery and is associated with worse postoperative outcomes (Jaber et al. 2021; Fineberg et al. 2014). The incidence of ileus after lumbar spine surgery has been reported across a wide range, between 2 and 33%, in the literature (Fineberg et al. 2014; Bahk et al. 2020; Kiely et al. 2016; Lee et al. 2015; Oh et al. 2015). This huge discrepancy in the reported incidence may stem from ambiguous definitions of postoperative ileus between different studies. Thus, an objective measure that sensitively reflects the spectrum of postoperative gastrointestinal (GI) impairment is clinically relevant to improve the quality of postoperative care.

Recently, the I-FEED classification, a 5-item scoring system of postoperative GI recovery, was proposed by the American Society for Enhanced Recovery and Perioperative Quality Initiative Joint Consensus Statement in 2018 to detect GI dysfunction after elective colorectal surgery (Hedrick et al. 2018). The benefits of the I-FEED classification for detecting postoperative GI impairment were twofold. First, postoperative GI recovery is multidimensional, including nausea, vomiting, intolerance to oral intake, inability to pass flatus or stool, and abdominal distension. Hence, the implication of the I-FEED classification could avoid the dichotomous yes-or-no definition to assess a patient’s GI recovery. Second, this scoring system has been recently validated in two studies with patients undergoing colorectal surgery, and these results not only indicated sufficient sensitivity to detect subtle changes in postoperative GI recovery but also revealed its correlation with clinical outcomes (Alsharqawi et al. 2020; Leung et al. 2021). However, its generalizability to lumbar spine surgery remains unclarified. Therefore, we conducted this prospective observational study to evaluate the validity of the I-FEED score classification for postoperative GI impairment in patients undergoing lumbar spine surgery.

## Material and methods

This study was approved by the research ethics committee (the Research Ethics Committee of National Taiwan University Hospital) and registered at clinical.gov (NCT04057599). After obtaining informed consent, adult patients undergoing elective lumbar spine surgery with general anesthesia were enrolled between August 2019 and February 2022. Baseline demographic and clinical data were collected at the time of enrollment. Patients with preoperative functional constipation diagnosed based on the ROME IV criteria (Aziz et al. 2020) were excluded, and surgical invasiveness was scored based on a validated index based on the number of vertebrae decompressed, fused, or instrumented (Mirza et al. 1976).

### Perioperative care

Each patient received general anesthesia that was maintained by using a combination of intravenous fentanyl and inhalation of sevoflurane to the anesthetic depth of a bispectral index between 40 and 60. The intraoperative mean arterial pressure was maintained at ≥ 60 mmHg by using norepinephrine infusion or iv. fluid challenge (Sessler et al. 2019). Blood transfusion was performed when the patient’s hemoglobin level was less than 9 g/dL.

Postoperative pain was managed by using the combination of oral tramadol/paracetamol (Dogar and Khan 2017), nonsteroidal anti-inflammatory drugs, and iv. morphine. The use of prophylactic ondansetron to prevent postoperative nausea and vomiting (PONV) was at the discretion of the attending anesthesiologist. The PONV was managed by using metoclopramide, and a laxative (rectal bisacodyl) may be administered for intolerable abdominal distention with failure to pass flatus for at least 24 h (Wiriyakosol et al. 2007).

### Study measures and construct validity assessment

Trained research nurses who did not participate in clinical care measured the I-FEED score for 4 days after surgery. Table 1 illustrates the I-FEED classification scheme, which incorporates five elements: intake (score: 0, 1, 3), feeling nauseated (score: 0, 1, 3), emesis (score: 0, 1, 3), results of physical exam (score: 0, 1, 3), and duration of symptoms (score: 0, 1, 2). The total score, ranging from 0 to 14, is calculated and summed for each postoperative day, and the highest overall score is used to categorize patients into one of three categories: normal (0–2 points), postoperative gastrointestinal intolerance (POGI; 3–5 points), and postoperative gastrointestinal dysfunction (POGD; 6 + points). These three categories have been applied in colorectal surgery, abdominal surgery, and gynecological surgery to provide a consistent and objective definition of postoperative gastrointestinal function, as well as the trajectory of postoperative gastrointestinal recovery (Alsharqawi et al. 2020; Leung et al. 2021; McLemore et al. 2022; Lu et al. 2021; Gungorduk et al. 2024). A brief explanation of the three categories is summarized below:

1. Normal (score 0–2): These patients tolerate a diet without symptoms of bloating but may experience transient PONV or mild abdominal distension within 24–48 h after surgery.
2. Postoperative gastrointestinal intolerance (POGI; score 3–5): These patients typically experience nausea, small-volume emesis, and bloating 48 h after surgery. However, the majority of these patients tolerated oral liquids.
3. Postoperative gastrointestinal dysfunction (POGD; score ≥ 6): This is the most severe form of postoperative GI impairment. These patients develop abdominal distention with tympany, nausea resistant to antiemetics, and large-volume bilious emesis.

**Table 1:** The I-FEED classification scheme

Scoring item	Intake	Feeling nauseated	Emesis	Exam	Duration of symptom
**Description (score)**	0: tolerating oral diet	0: none	0: none	0: no abdominal distension	0: within 24 h
	1: limiting tolerance	1: responsive to treatment	1: one or more episodes of nonbilious emesis less than 100 ml	1: distension without tympany	1: 24–72 h
	3: complete intolerance	3: resistant to treatment	3: one or more episodes of emesis less than 100 ml or bilious emesis	3: significant distension with tympany	2: > 72 h
**Total score**	Score 0–2, normal; score, 3–5 postoperative gastrointestinal intolerance (POGI); score ≥ 6, postoperative gastrointestinal dysfunction (POGD)				

Because there is no gold standard definition for ileus after lumbar spine surgery, this study aimed to perform construct validity hypothesis testing, which determines the degree to which the I-FEED score is consistent with objective clinical findings relevant to gastrointestinal impairment, which are listed below:

1. Patients classified as POGD required a longer length of hospital stay (Hedrick et al. 2018).
2. Patients classified as POGD required a higher medical cost (exclusion of operation-related costs) (Fineberg et al. 2014).
3. Patients with normal I-FEED scores (0–2) required GI medical treatment (Alsharqawi et al. 2020).
4. Patients classified as POGD had a higher incidence of postoperative non-GI complications defined by using the International Statistical Classification of Diseases and Related Health Problems 10th Revision (ICD-10) (Hedrick et al. 2018; Alsharqawi et al. 2020).

Since the present study aimed to validate the I-FEED scoring system, the treatment for postoperative gastrointestinal impairment was not tailored based on the three categories to avoid interfering with the validation process.

### Statistical analysis

The sample size calculation was guided by previous studies, as the power calculation is not reliable for correlation analysis, but it is recommended that studies investigating construct validity and responsiveness should ideally include at least 100 patients (Terwee et al. 2012). Data are presented as the mean (standard deviation, SD), median (interquartile range, IQR), or number (%). Comparisons for dichotomous variables were performed using *χ*^2^. Continuous variables were compared using ANOVA or the Kruskal–Wallis test. All analyses were performed using MedCalc version 20.110 (MedCalc Software, Ostend, Belgium).

## Results

There were a total of 156 patients enrolled in this study. The patient characteristics and perioperative profile are listed in Table 2. Based on the I-FEED classification, 39 patients (25.0%) were classified as normal, 77 patients (49.4%) were classified as POGI, and 40 patients (25.6%) were classified as POGD. There were more male patients with normal I-FEED scores (61.5% vs. 44.2% vs. 32.5%; *p* = 0.033). Additionally, a greater proportion of patients in the normal and POGI categories had diabetes compared to those in the POGD category (30.8% vs. 22.2% vs. 2.5%; *p* = 0.004). The age, body mass index, and comorbidities, including cardiac disease, chronic obstructive lung disease, and cerebral disease, were comparable between the three patient groups.

**Table 2:** Patient characteristics of the study population

	Overall (*n* = 156)	Normal (*n* = 39)	POGI (*n* = 77)	POGD (*n* = 40)	*p*-value
Age	61.1 ± 13.5	58.8 ± 14.8	61.6 ± 14.1	62.1 ± 10.9	0.482
Male (n; %)	71 (45.5%)	24 (61.5%)	34 (44.2%)	13 (32.5%)	0.033
Body mass index (kg/m^2^)	25.7 ± 3.4	25.7 ± 3.7	25.9 ± 3.5	25.5 ± 3.1	0.843
Cardiac disease	33 (21.2%)	11 (28.2%)	17 (22.1%)	5 (12.5%)	0.223
COPD	3 (1.9%)	1 (2.6%)	2 (2.6%)	0 (0%)	0.948
Smoking	16 (10.3%)	4 (10.3%)	8 (10.4%)	4 (10.0%)	0.998
Diabetes	30 (19.2%)	12 (30.8%)	17 (22.1%)	1 (2.5%)^#*^	0.004
Cerebral disease	9 (5.8%)	3 (7.7%)	5 (6.5%)	1 (2.5%)	0.570
Surgical type					
Decompression (n; %)	32 (20.5%)	11 (28.2%)	15 (19.5%)	6 (15.0%)	0.264
Fusion (n; %)	105 (67.3%)	21 (53.8%)	52 (67.5%)	32 (80.0%)	
HIVD (n; %)	18 (11.6%)	7 (18.0%)	9 (11.7%)	2 (5.0%)	
Scoliosis (n; %)	1 (0.6%)	0 (0%)	1 (1.3%)	0 (0%)	
Surgery etiology					
Deformity (n; %)	2 (1.3%)	1 (2.6%)	1 (1.3%)	0 (0%)	0.513
Degeneration (n; %)	148 (94.8%)	36 (92.3%)	72 (93.5%)	40 (100%)	
Trauma (n; %)	2 (1.3%)	0 (0%)	2 (2.6%)	0 (0%)	
Tumor (n; %)	4 (2.6%)	2 (5.1%)	2 (2.6%)	0 (0%)	

*COPD* chronic obstructive pulmonary disease, *POGI* postoperative gastrointestinal intolerance, *POGD* postoperative gastrointestinal dysfunction

^#^indicates a *p*-value < 0.05 compared to that of normal I-FEED score; ^*^indicates a *p*-value < 0.05 compared to that of POGI

Table 3 summarizes the intraoperative profiles. The intraoperative profile, including the spine surgical invasiveness, operation time, anesthetic time, blood loss, transfusion, prophylactic antiemetic use, norepinephrine dose, and opioid requirement, was comparable between patients in the three I-FEED categories. Table 4 presents the details of the I-FEED scores in the three groups. Among the whole cohort, the three most common GI impairment presentations based on the I-FEED classification were as follows: abdominal distension without tympanic percussion (85.2%), limited tolerance to oral intake (73.7%), and nausea responsive to treatment (49.4%). The proportions of patients with limited tolerated oral intake were comparable between patients classified as POGI and POGD but were significantly higher than those who were classified as normal (10.3% vs. 92.2% vs. 100%; *p* < 0.001). The proportions of patients with nausea responsive to treatment (scored one) were significantly different between the three I-FEED groups (0% vs. 54.5% vs. 87.5%; *p* < 0.001). There was a high proportion of patients (31/40; 77.5%) classified as POGD who had episodes of high volume (> 100 ml) emesis, but no patients with normal I-FEED experienced emesis. By comparison, the presentation of abdominal distension without tympany (scored one) was prevalent among the entire cohort (approximately 86%), and there were comparable proportions of patients as the POGI (11.7%) and POGD (15.0%) with significant distension with tympany (scored 3). Regarding the duration of GI impairment symptoms, only patients with POGI and POGD revealed prolonged symptoms more than 72 h after surgery.

**Table 3:** Intraoperative profiles

	Overall (*n* = 156)	Normal (*n* = 39)	POGI (*n* = 77)	POGD (*n* = 40)	*p*-value
Surgical invasiveness	7.8 ± 5.6	6.3 ± 5.3	8.0 ± 5.9	8.7 ± 5.0	0.134
Operation time (min)	133 ± 70	130 ± 74	135 ± 73	134 ± 61	0.932
Blood loss (ml)	100 (20–300)	20 (20–275)	100 (20–300)	125 (20–325)	0.153
Norepinephrine dose (μg)	33.5 ± 135.7	12.3 ± 30.3	47.1 ± 183.5	29.6 ± 76.1	0.416
Transfusion (n; %)	21 (13.5%)	4 (10.3%)	9 (11.7%)	8 (20.0%)	0.364
Prophylactic ondansetron (n; %)	19 (12.2%)	4 (10.3%)	11 (14.3%)	4 (10.0%)	0.731
Fentanyl dose (μg)	129 ± 49	119 ± 33	136 ± 52	124 ± 53	0.178

*POGI* postoperative gastrointestinal intolerance, *POGD* postoperative gastrointestinal dysfunction

**Table 4:** Details of the I-FEED classification score

	Overall (*n* = 156)	Normal (*n* = 39)	POGI (*n* = 77)	POGD (*n* = 40)	*p*-value
Intake					*p* < 0.001
0 (n; %)	41 (26.3%)	35 (89.7%)	6 (7.8%)	0 (0%)	
1 (n; %)	115 (73.7%)	4 (10.3%)	71 (92.2%)^*^	40 (100%)^*^	
3 (n; %)	0 (0%)	0 (0%)	0 (0%)	0 (0%)	
Feeling nauseated					*p* < 0.001
0 (n; %)	79 (50.6%)	39 (100%)	35 (45.5%)	5 (12.5%)	
1 (n; %)	77 (49.4%)	0 (0%)	42 (54.5%)^*^	35 (87.5%)^#*^	
3 (n; %)	0 (0%)	0 (0%)	0 (0%)	0 (0%)	
Emesis (n; %)					*p* < 0.001
0 (n; %)	98 (62.8%)	39 (100%)	55 (71.4%)	4 (10.0%)	
1 (n; %)	27 (17.3%)	0 (0%)	22 (28.6%)	5 (12.5%)	
3 (n; %)	31 (19.9%)	0 (0%)	0	31 (77.5%)^#*^	
Exam (n; %)					*p* < 0.001
0 (n; %)	8 (5.1%)	7 (17.9%)	1 (1.3%)	0 (0%)	
1 (n; %)	133 (85.2%)	32 (82.1%)	67 (87.0%)	34 (85.0%)	
3 (n; %)	15 (9.6%)	0 (0%)	9 (11.7%)^*^	6 (15.0%)^*^	
Duration of symptoms					*p* < 0.001
0 (n; %)	54 (34.6%)	20 (51.3%)	16 (20.8%)	18 (45.0%)	
1 (n; %)	87 (55.8%)	19 (48.7%)	57 (74.0%)	11 (27.5%)	
2 (n; %)	15 (9.6%)	0 (0%)	4 (5.2%)	11 (27.5%)	

*POGI* postoperative gastrointestinal intolerance, *POGD* postoperative gastrointestinal dysfunction

^*^Indicates *p* < 0.05 compared to the normal I-FEED score. ^#^Indicates *p* < 0.05 compared to that of the POGI

Table 5 summarizes the postoperative profiles. Patients with higher I-FEED scores agreed with the four validity hypotheses. Patients with POGD had a significantly longer length of hospital stay [4 (3–5) vs. 5 (4–7) vs. 5 (4–7) days in the normal, POGI and POGD groups, respectively; *p* = 0.049], more inhospital medical costs [806 (582–1633) vs. 1238 (740–1988) vs. 1319 (907–2303) Taiwanese dollars in the normal, POGI and POGD groups, respectively; *p* = 0.037], and more patients with POGD required rectal laxative (10.3% vs. 32.5% vs. 32.5% in the normal, POGI and POGD groups, respectively; *p* = 0.026) than those with normal I-FEED scores. In addition, more patients with POGD had non-GI complications than patients in the other two groups (5.1% vs. 11.7% vs. 30.0%; *p* = 0.034). The perioperative morphine consumption was comparable between the three I-FEED groups, but although patients with POGD exhibited higher tramadol utilization, this increase did not achieve statistical significance (*p* = 0.077). Moreover, despite patients with POGD requiring a greater amount of perioperative intravenous fluid administration, this difference also failed to attain statistical significance (1661 ± 1012 ml vs. 2183 ± 1433 ml vs. 2334 ± 1163 ml in the normal, POGI and POGD groups, respectively; *p* = 0.052).

**Table 5:** Postoperative outcomes

	Overall (*n* = 156)	I-FEED 0–2 (*n* = 39)	I-FEED 3–5 (*n* = 77)	I-FEED ≧ 6 (*n* = 40)	*p*-value
Length of hospital stay (day)	5 (3–6)	4 (3–5)	5 (4–7)	5 (4–7)^*^	0.049
Medical cost for GI symptoms (TW dollar)	1204 (710–1951)	806 (582–1633)	1238 (740–1988)	1319 (907–2303)^*^	0.037
Laxative requirement (*n*; %)	42 (6.9%)	4 (10.3%)	25 (32.5%)^*^	13 (32.5%)^*^	0.026
Non-GI complication (*n*; %)	17 (10.9%)	1 (2.6%)	7 (9.1%)	9 (22.5%)	0.014
Perioperative morphine consumption (mg)	7.7 ± 8.4	6.7 ± 5.9	7.9 ± 8.1	8.2 ± 10.9	0.703
Perioperative tramadol consumption (mg)	112.7 ± 190.2	67.3 ± 154.4	116.7 ± 192.1	149.1 ± 212.5	0.077
Perioperative iv. fluid (ml)	1984 ± 1281	1661 ± 1012	2183 ± 1488	2334 ± 1163	0.052

*LOS* length of hospital stay, *TW* Taiwanese, *GI* gastrointestinal

^*^Indicates *p* < 0.05 compared to patients with an I-FEED score of 0–2

## Discussion

This is the first prospective study to investigate the validity of the I-FEED classification to detect postoperative GI impairment in noncolorectal surgery. We observed potentially adequate validity of this scoring system for assessing postoperative GI impairment after lumbar spine surgery.

The incidence of postoperative ileus after lumbar spine surgery was reported to be between 3 and 15% in the retrospective literature (Jaber et al. 2021; Fineberg et al. 2014; Bahk et al. 2020; Kiely et al. 2016). However, we observed a higher incidence of POGD (approximately 25%). This discrepancy may be due to several reasons. First, it may occur that only the most severe form of bowel obstruction was documented in records in case no daily assessment of GI impairment based on consistent criteria was applied. Second, there is a lack of consistency between various definitions of ileus in the literature. Accordingly, Wolthuis et al. (2016) conducted a systemic review for ileus after colorectal surgery and indicated that there were five different definitions of ileus, and these discrepancies in definition resulted in a wide range of incidence between 2.3 and 61%. Third, retrospective analysis based on the billing code is occasionally associated with lower sensitivity to detect real inhospital pathology (Grams et al. 2014). Therefore, an objective measure with sufficient sensitivity to detect postoperative GI impairment in lumbar spine surgery is highly warranted. Recently, Oh et al. (2015) reported that 32% of patients developed radiographic paralytic ileus after lumbar spine surgery which was close to the incidence of POGD in our cohort. Abdominal plain radiography is sensitive for detecting abdominal obstruction (Kim et al. 2011), but it is associated with more medical costs, and more experienced radiologists are required for evaluation (Thompson et al. 2007). By comparison, the I-FEED classification could be performed at the bedside, and this test required minimal physical examination skills that were familiar to most care providers.

In the present study, we observed an acceptable construct validity of the I-FEED classification, as patients classified as POGD were associated with worse postoperative outcomes related to GI impairment, which was compatible with the original proposal applied to patients undergoing colorectal surgery (Hedrick et al. 2018). Specifically, a higher I-FEED score was associated with a longer hospital stay and more laxative treatment. The I-FEED classification was also devised to reflect the increased cost related to GI impairment (Hedrick et al. 2018). In this study, we observed that patients with higher I-FEED scores required higher inhospital medical costs. Furthermore, the original proposal of the I-FEED-defined POGD was to identify patients with higher risks of postoperative non-GI complications (Hedrick et al. 2018). The postoperative complication rate of the present study (10.9%) was similar to that rate in a recent European report with a larger sample size (15.3–22.3%) (Barbanti-Brodano et al. 2020). We further observed that more than half of the patients who developed non-GI complications were classified as POGD by the I-FEED classification. These associations revealed the I-FEED scoring system as a potentially reliable measure to represent both perioperative GI dysfunction and related clinical burden.

Several risk factors for postoperative ileus were reported in previous retrospective reports, including higher surgical complexity (spine surgical invasiveness), higher blood loss (Fineberg et al. 2014; Bahk et al. 2020), higher iv fluid amount (Fineberg et al. 2014; Kiely et al. 2016), and higher opioid consumption (Gifford et al. 2019). Intraoperative spinal manipulations over upper lumbar levels may damage splanchnic nerves, resulting in decreased GI motility, and hence, higher spine surgical invasiveness may be harmful to postoperative GI motility (Boos and Aebi 2008). Accordingly, surgery with a higher invasiveness score is associated with more blood loss and may influence GI motility more. Increased iv. fluid amount may result in edema, and it has been frequently reported to be related to postoperative ileus in robotic surgery, colorectal surgery, and lumbar spine surgery (Kiely et al. 2016; Koch et al. 2021; VandeHei et al. 2017; Shim et al. 2021). Regarding opioid consumption, oral tramadol may also increase the risk of PONV (Liukkonen et al. 2002). In the present study, we observed a high incidence of PONV (feeling nauseated and emesis). The PONV incidence after lumbar spine surgery has been reported to be more than 50% despite prophylactic 5-HT_3_ antagonist treatment (Roh et al. 1976). In this study, we accordingly observed that the prophylactic 5-HT_3_ antagonist was not protective against the development of POGD, and one-third of patients required postoperative antiemetics. This is also compatible with a previous report indicating that prophylactic medication was ineffective in preventing ileus after lumbar spine surgery (Oh et al. 2015). Furthermore, we observed a high incidence of postoperative abdominal distension (approximately 86%) in this study. This is compatible with the recently reported rate of abdominal pain (71%) after lumbar spine surgery in a Korean cohort (Bahk et al. 2020). However, this symptom does not necessarily increase the clinical burden, but the I-FEED classification may be helpful for clinicians to detect clinically relevant risk stratification.

There were limitations in this study. First, this is a single-center study with relatively small numbers of patients. Therefore, this study may be underpowered to detect risk factors of POGD such as the spine surgery invasiveness, blood loss, perioperative iv. fluid amount, and the opioid consumptions. However, the good sensitivity of the I-FEED classification was sufficiently observed. Second, we did not propose a treatment protocol based on the I-FEED score for the management of GI impairment after lumbar spine surgery. It remains unclear whether the I-FEED scoring system could reflect the treatment effects. Third, the “duration of symptoms” item of the I-FEED classification is not scored on a daily basis, as the scored item contains a 3-day time frame. As we observed that more than half of the patients in our cohort had one score in the duration item, we expect that expansion of this category to more subunits may also extend the accuracy of I-FEED classification for risk stratification of patients undergoing lumbar spine surgery.

## Conclusion

In summary, we indicated preliminary construct validity evidence for the I-FEED as a measure of postoperative GI impairment in patients undergoing elective lumbar spine surgery. This is the first validated data to support I-FEED other than colorectal surgery. The treatment protocol based on the I-FEED classification may be developed in further studies.

## Data Availability

The datasets used and/or analyzed during the current study are available from the corresponding author on reasonable request.

## Abbreviations

POGI: Postoperative gastrointestinal intolerance
POGD: Postoperative gastrointestinal dysfunction
PONV: Postoperative nausea and vomiting
